# Phase I and Phase II Therapies for Acute Ischemic Stroke: An Update on Currently Studied Drugs in Clinical Research

**DOI:** 10.1155/2017/4863079

**Published:** 2017-02-14

**Authors:** Cesar Reis, Onat Akyol, Wing Mann Ho, Camila Araujo, Lei Huang, Richard Applegate II, John H. Zhang

**Affiliations:** ^1^Department of Anesthesiology, Loma Linda University Medical Center, Loma Linda, CA 92354, USA; ^2^Department of Physiology and Pharmacology, Loma Linda University School of Medicine, 11041 Campus Street, Risley Hall, Room 219, Loma Linda, CA 92354, USA; ^3^Department of Neurosurgery, University Hospital Innsbruck, 6020 Tyrol, Austria; ^4^Department of Neurosurgery, Loma Linda University School of Medicine, Loma Linda, CA 92354, USA

## Abstract

Acute ischemic stroke is a devastating cause of death and disability, consequences of which depend on the time from ischemia onset to treatment, the affected brain region, and its size. The main targets of ischemic stroke therapy aim to restore tissue perfusion in the ischemic penumbra in order to decrease the total infarct area by maintaining blood flow. Advances in research of pathological process and pathways during acute ischemia have resulted in improvement of new treatment strategies apart from restoring perfusion. Additionally, limiting the injury severity by manipulating the molecular mechanisms during ischemia has become a promising approach, especially in animal research. The purpose of this article is to review completed and ongoing phases I and II trials for the treatment of acute ischemic stroke, reviewing studies on antithrombotic, thrombolytic, neuroprotective, and antineuroinflammatory drugs that may translate into more effective treatments.

## 1. Introduction

Almost 2 decades after the demonstration of a decrement and in some instance absence of disability and the consequent approval of r-tPA for treatment of acute ischemic stroke (AIS), a plethora of research has been performed to better understand not only the mechanisms involved in protecting against AIS but also the synergy that different drugs produce in AIS treatment. After many years, a growing number of ischemic stroke patients lack other treatment options. A number of phase I and phase II clinical trials designed to develop better strategies to treat AIS are currently in progress or completed.

**Figure 1:**
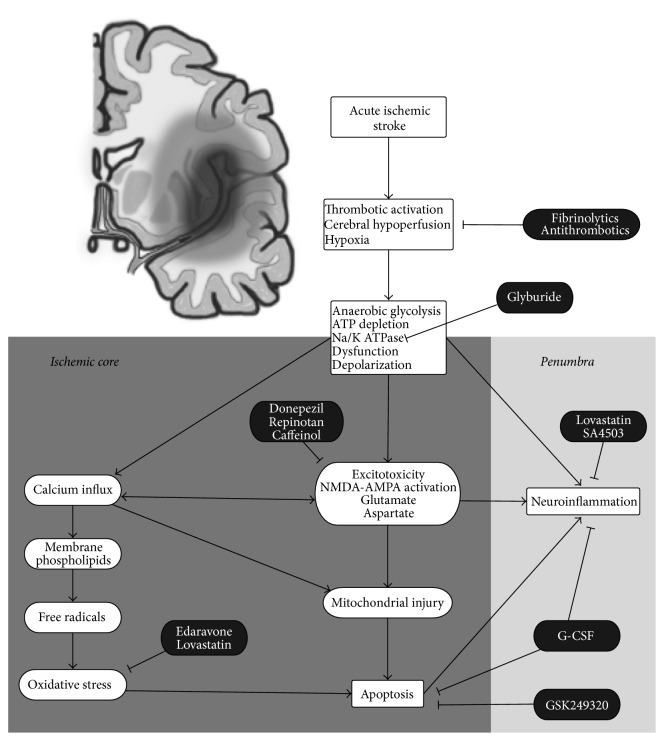
This figure provides an overview of the cellular mechanisms activated after cerebral ischemia and their respective targets by the different therapies (excitotoxicity and glutamate release, neuroinflammation with proinflammatory and anti-inflammatory states, etc.).

The understanding of ischemic stroke pathophysiological mechanisms is expanding. Comprehensive research in drug development builds upon experimental ischemic stroke models to recognize the mechanisms that underlie cerebral ischemic injury. The lack of oxygen results in energy deprivation and the ischemic cascade starts with an arterial thromboembolic episode. The main aim of phases I and II clinical trials in AIS is to rescue and restore the ischemic penumbra within a specified therapeutic window. Otherwise, the abrogation of energy weakens ion homeostasis and provokes a rise in the extracellular concentration of K^+^, along with a decline in extracellular concentrations of Na^+^ and Cl^−^. This anoxic depolarization triggers not only the formation of reactive oxygen species, glutamate release, and dysregulation in intracellular Ca^2+^ levels, but also mitochondrial membrane collapse and induction of neuroinflammation [1]. Expeditious recanalization is mandatory to avoid an ischemic cascade that generates neuronal tissue infarction. There are drugs in development that aim at inducing possible neuroprotective factors and/or pathways, facilitating immediate reperfusion to alleviate the ischemic injury, blocking platelet aggregation and coagulation, and degrading fibrin. Neuroprotective agents protect ischemic neurons in the acute phase of the ischemic stroke. We will provide an overview of the cellular mechanisms activated after cerebral ischemia and their respective targets in Figure 1.

## 2. Antithrombotic Drugs

### 2.1. Eptifibatide

Eptifibatide is an antiplatelet drug belonging to the glycoprotein IIb/IIIa inhibitor class. It binds to glycoprotein IIb/IIIa in between the IIb and IIIa arms of the activated platelet, effectively blocking the binding domain from fibrinogen, thus inhibiting thrombi formation. It has been studied in combination with aspirin, low molecular weight heparin (tinzaparin), and intravenous (i.v.) r-tPA therapy in several dosing regimens, since r-tPA alone is inadequate to recanalize large arterial occlusions in approximately 50% of cases. In recent years, three trials have tried to establish safety of eptifibatide and they all kept their inclusion criteria constant [2].

The CLEAR trial was designed to establish the safety of eptifibatide in combination with r-tPA in the treatment for AIS. The endpoint was within 36 hours, finding that a dose escalation combination of reduced-dose r-tPA plus eptifibatide would justify further dose-ranging trials in AIS [3]. One group received low-dose r-tPA with eptifibatide (75 ug/kg bolus and 0.75 ug/kg/min infusion over 2 hours) and the other group received the standard dose of r-tPA. The primary safety endpoint was defined as the incidence of a symptomatic intracerebral hemorrhage (sICH) within the period of 36 hours. Even though this trial found promising results and was a randomized, blinded, safety trial measuring low-dose r-tPA plus eptifibatide, it had a marked disparity in age and baseline NIH Stroke Scale/Score (NIHSS) between the combination therapy and the control groups [3]. Higher age and NIHSS are predictors of sICH after r-tPA treatment following AIS [4]. On the other hand, the study not only had the EuroQoL quality of life index and Stroke-Specific Quality of Life Scale administered at 3 months, but also has a 90-day outcome assessed by certified investigators in the NIHSSS. Additionally, they received standardized training regarding the modified Rankin Scale (mRS), Barthel, Glasgow, and EuroQol assessments. Furthermore, the study was not associated with acute treatment of patients, which brings more validity to the outcome score obtained. In light of these points, the CLEAR trial offered insights and hope for well-designed studies in the future in order to research the effects of this combination using a dose escalation regimen.

Following the CLEAR trial, investigators conducted the CLEAR-ER trial, a multicenter, double-blinded, randomized safety study that enrolled a total of 126 AIS patients (NIHSS score > 5) having mRS score as the primary efficacy outcome measurement. The CLEAR-ER trial added extra points to measure not only early patient improvement at 2, 24 hours, and 90 days per NINDS investigators' suggestion, but also systemic bleeding at 7 days after therapy. This trial consolidated the safety of combination therapy even though mild bleeding was found in the combination group (no interventions were necessary) and the sICH rate in the r-tPA group was greater than expected. A larger trial is necessary to address sICH differences from CLEAR-ER when compared to NINDS trials. However, combination treatment not only proved to be safe when given within 3 hours of symptom onset [5] but additionally proved to be realistic to pursue translation with combination of eptifibatide.

After the aforementioned trials were published, a full dose regimen trial was designed. CLEAR-FDR was a single-arm, prospective, open-label, multisite study using 0.9 mg/kg i.v. r-tPA within 3 hours of symptom onset followed by eptifibatide (135 *μ*g/kg bolus and 2-hour infusion at 0.75 *μ*g/kg per minute). A repeat NIHSS score was obtained at the end of the 2-hour eptifibatide infusion and at 24 (±6) hours after r-tPA. 27 patients were enrolled in this trial, a number at least 3x less than the amount enrolled in the CLEAR trial and almost 5x less than the CLEAR-ER trials. Importantly the single-arm nonblinded study designs with enrollment by a single regional stroke team are also limiting factors [6].

**Figure 2:**
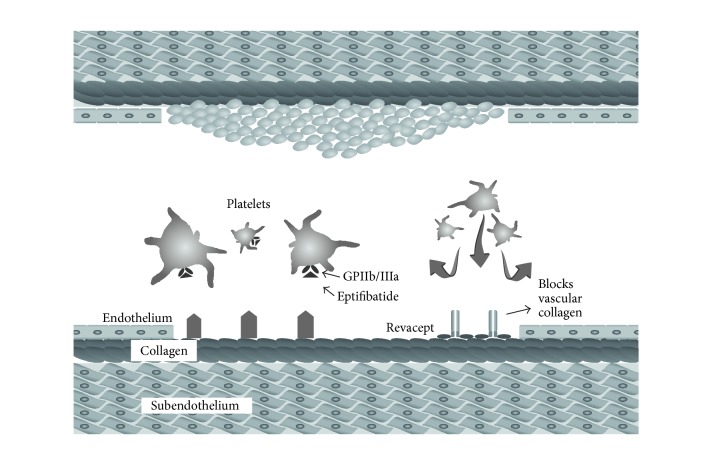
The drugs eptifibatide and revacept act by decreasing platelet aggregation and further formation of a clot.

Aside from the limitations of the CLEAR-FDR, there is a plan to investigate the dose response of r-tPA plus eptifibatide via a pooled analysis of all 3 completed trials before moving on to a phase III clinical trial. This will help estimate the variability of the trials. A demonstration of eptifibatide's mode of action is found in Figure 2.

### 2.2. Revacept

Revacept is a dimeric glycoprotein VI-fc that blocks glycoprotein VI-dependent pathways. By interfering with the vascular collagen site, it blocks vascular collagen in plaques or exposed by erosion thus reducing platelet adhesion. It was found to be safe in preclinical studies. Thirty healthy men received a single i.v. administration of 10, 20, 40, 80, or 160 mg revacept in a phase I study [7] that evaluated the pharmacological parameters of the drug itself. Different from the CLEAR trials, its inclusion criteria were more selective to nonsmoking white men between the ages of 18–35, normotensive, and with body weight ranging from 75 to 85 kg. The concentration of revacept plasma was found to interrupt aggregation beginning 2 hours after the administration of the drug and produced significant inhibition 24 hours and 7 days following infusion with higher doses. Bleeding time was not significantly affected, and ADP (thrombin receptor activating peptide) dependent platelet aggregation was not changed. The drug's effect was longer lasting in humans than in previously conducted animal studies, which is likely due to the longer half-life. Among the pros and cons of the study, revacept was safe and well tolerated in a dose dependent pharmacologic profile.

Revacept is also being studied in conditions associated with stroke, such as carotid stenosis, that presents with microembolic signals (MES) [8]. MES are frequently found in patients with acute stroke. An ongoing phase II trial using a 20-minute single dose of revacept plus antiplatelet monotherapy (aspirin or clopidogrel) or monotherapy alone aims to reduce MES. Figure 2 demonstrates revacept action on decreasing platelet aggregation formation of a clot.  Table 1 summarizes details of completed clinical trials testing these antithrombotic drugs.

## 3. Thrombolytic Drugs

### 3.1. Reteplase

Reteplase is a nonglycosylated deletion mutein of tPA, similar to alteplase but modified in order to achieve a longer half-life (approximately 13–16 minutes), as well as improved thrombolytic properties by binding to fibrin with a lower affinity than alteplase.

A randomized, feasibility study using primates demonstrated preliminary support that IA (intra-arterial) reteplase with IV abciximab, as well as IA reteplase without IV abciximab, was effective in obtaining recanalization in an intracranial thrombosis model [27]. A prospective, nonrandomized, open-label trial was conducted to evaluate the safety of an escalating dose of reteplase in conjunction with i.v. abciximab in patients with AIS (3–6 h after symptom onset). The authors hypothesized higher rates of recanalization and improved clinical outcomes were due to the combination of medications that lyse fibrin and prevent aggregation of platelets [9]. Patients had NIHSS scores of 4 or greater or 23 or less and arterial occlusion demonstrated by diagnostic angiography. Partial or complete recanalization was observed in 13 of the 20 patients. Thirteen patients demonstrated early neurological improvement, and favorable outcome at 1 month was observed in six patients. In this study, a combination of intra-arterial reteplase and i.v. abciximab was safely administered to patients with ischemic stroke presenting between 3 and 6 hours after symptom onset.

Reteplase was first studied in a primate model of intracranial thrombosis [27], but the study was nonrandomized and did not meet RIGOR [28] and STAIR criteria. RIGOR and STAIR criteria are a set of recommendations for performing better research, including the encouragement of randomization, blinded studies, power analyses, and the like [28].

### 3.2. Tenecteplase

Tenecteplase is a genetically engineered mutant tPA which may possess some advantages over alteplase, such as a longer half-life, more resistance to plasminogen activator inhibitor, more fibrin specificity, and producing less systemic depletion of circulating fibrinogen. These advantages lead to faster perfusion and lower incidences of sICH [8]. In a New Zealand study, Parsons et al. set out to determine whether alteplase or tenecteplase had superior outcomes. In this phase IIb trial, 75 AIS patients received treatment in the form of alteplase (0.9 mg/kg) or tenecteplase, low dose (0.1 mg/kg) or high dose (0.25 mg/kg). The baseline NIHSS scores of all patients are approximately 14 (+−2.6). Treatment was administered within 3-4 hours of stroke onset. Outcome measures included the percentage of perfusion of the lesion measured by MRI, clinical and neurological improvements measured by a change in NIHSS score, and changes in mRS scores at 24 hours. There was a significant improvement in the tenecteplase groups, with 79% average reperfusion, compared to 55% in the alteplase group, as well as 64% of tenecteplase group patients having a reduced NIHSS score by 8 or greater, compared to 36% of the alteplase group. The authors conclude that phase III trials are appropriate based on their findings [10]. A Scottish study also tested tenecteplase versus alteplase with the endpoint being the percentage of penumbra salvaged seen via CT at 24–48 hours after treatment. In a phase II, prospective, randomized, open-label, blinded study, 104 patients were treated with the standard dose of alteplase or high dose (0.25 mg/kg) of tenecteplase within 4.5 hours of stroke onset. Of the 104 patients enrolled, 35 tenecteplase and 36 alteplase patients contributed to the endpoint. No difference was found between the groups in salvaged penumbra, incidences of sICH, or other adverse effects [11].  Table 2 summarizes details of completed clinical trials testing these thrombolytic drugs.

## 4. Endovascular Procedure Trials

The advantages of intra-arterial treatment in AIS with anterior large-vessel occlusion were proven in many randomized controlled trials (RCTs) and consolidated into clinical practice. Acute large-vessel occlusions have less benefit from r-tPA treatment. Critical time points during intra-arterial treatment include time to angiography/perfusion imaging and time to start revascularization with r-tPA in order to get improved neurologic outcomes. There are 5 prospective randomized open blinded endpoint- (PROBE-) designed trials published between 2014 and 2015 in acute large-vessel anterior circulation ischemia and they focus on 90-day outcomes. According to results of these trials, endovascular treatment groups have higher outcome scores (mRS 0–2) as a primary endpoint. These 5 trials are the following.

ESCAPE had 315 patients studied with multiphase CTA to determine the intracranial collateral circulation. The start of CT to groin puncture was less than 60 minutes and time to first reperfusion was less than 90 minutes. Different from other RCTs, the time window in the study was 12 hours; however the study was not powered to evaluate endovascular therapy between 6 and 12 hours after symptom onset because of insufficient patient number [8].

Campbell BC and colleagues published the EXTEND-IA trial. The study was stopped early because of efficacy at 70 patients. Using advanced penumbral imaging techniques, control patients had lower median NIHSS scores compared to the endovascular group (13 versus 17, resp.). The other 4 RCTs mentioned in this section have used ASPECT scoring for infarct region, which was not used by the EXTEND-IA trial. This trial focused on patients with ischemic core volume of less than 70 mL who received i.v. r-tPA within 4.5 hours' time window. Similar to the ESCAPE trial, internal carotid artery or proximal MCA occluded patients were enrolled [29].

The SWIFT PRIME trial had an 88% rate of complete reperfusion, the highest rate in comparison to the other RCTs mentioned in this section. This finding may be associated with exclusion of patients with extra cranial carotid occlusion. Only EXTEND-IA and SWIFT PRIME trials have 100% of their active control groups receiving i.v. r-tPA and comparing them to intra-arterial recanalization with i.v. r-tPA in the study groups [30].

In October 2015, REVASCAT was published. It had the longest median time period of 269 minutes up to recanalization. Patients with confirmed revascularization after r-tPA were excluded from this study. Patients who have failed treatment for large-vessel occlusion after r-tPA, confirmed on computed tomographic angiography, were the focus of endovascular treatment. This trial has the lowest sICH rates among the other 4 endovascular trials, further supporting the efficacy of endovascular treatment up to 8 hours; however the study was terminated before completing planned enrollment [31].

A 2015 meta-analysis sought to compare outcomes in the form of functional independence (mRS scores between 0 and 2 at 90 days) in 8 clinical trial studies, including SYNTHESIS, MR RESCUE, MR CLEAN, ESCAPE, EXTEND-IA, and REVASCAT. Endovascular therapy treatment patients had better outcomes across the board. There were no significant differences in mortality or incidence of sICH between endovascular therapy groups and medication only groups. Authors concluded that the best course of action is a treatment that combines standard medical treatments with endovascular therapy for best outcomes for qualifying patients [32].

## 5. Neuroprotection

### 5.1. Lovastatin

Beside cholesterol reducing effects, statins are considered to have favorable impact on blood brain barrier, oxidative stress, cerebral blood flow, and inflammation according to previous studies. Experimental studies showed several statins have neuroprotective effects on neuronal injury and infarct size in rodent models of AIS when given both before and after AIS [33, 34]. However, a meta-analysis in 2011 by Squizzato et al. demonstrated that in 8 randomized clinical trials involving 625 participants statin treatment did not reduce all-cause mortality compared with placebo or no treatment in the 431 patients enrolled in 7 out of the 8 studies. This was explained as due to inadequate data [31].

The Neuroprotection with Statin Therapy for Acute Recovery Trial (NeuSTART) is a nonrandomized, single group assignment, phase I B dose-escalation study focused on testing the hypothesis that short-term statin therapy at maximally effective doses provides neuroprotection based on animal studies. In this trial AIS patients were treated within 24 hours of symptom onset. The maximum tolerated lovastatin dose was 8 mg/kg/day, which is a higher dose than currently approved by the FDA, and they found 13% toxicity. No clinical liver disease, myopathy, or creatine phosphokinase elevations were reported. This trial showed an appropriate treatment period of 3 days after an AIS for that dose. They found a significant decrease in TNF-*α* receptor 1 (TNFR1) levels associated with dose increase but no effects on CRP, IL-6, or TNF levels. In addition, no significant dose-related effect on platelet aggregation was detected. The limitations of this trial include insufficient patient number and no neurological outcome reported during dose escalation. On the other hand, it is encouraging to see that a dose higher than the current oral maximum was tolerated with low toxicity. Thus, this dose could be used in future placebo-controlled randomized trials for various outcomes [35].

Researchers are recruiting individuals for a phase II clinical trial with low and high dose lovastatin comparing to placebo within 24 hours of symptom onset. The inclusion criteria for this trial differ from the prior trial in that patients will receive both standard dose i.v. t-PA and/or mechanical interventional procedures in their respective groups. The study will focus primarily on musculoskeletal and hepatic toxicity with a 3-month follow-up period. Secondary targets are neurological outcomes and effects on inflammatory markers [14, 35].

### 5.2. Donepezil

Donepezil is a reversible, selective acetylcholinesterase inhibitor broadly used in the treatment of Alzheimer's dementia [36]. Enhancement of the cholinergic system showed beneficial effects in trials of chronic stroke [37, 38] and poststroke recovery [39–42] and in experimental stroke models [43]. These results led to the open-label study conducted by the Mayo Acute Stroke Trial for Enhancing Recovery (MASTER) Study Group. Thirty-three patients with AIS were treated with donepezil within 24 hours after event. Donepezil was demonstrated to be safe and tolerated at an initial dose of 5 mg daily for the first 4 weeks; then it was increased to 10 mg per day. Neurologic, cognitive, functional, and psychological outcomes 90 days after stroke with donepezil treatment compared to the National Institutes of Health Stroke Scale (NINDS) r-tPA trial data showed a tendency for favorable outcomes [44]. The evidence was satisfying enough for this research group to plan further investigation of donepezil in AIS management in a randomized study.

Limitations to the MASTER Study include the fact that it was single-armed and open-label study conducted with 33 patients and only 76% of the enrolled patients completed the 90-day treatment. There was no concurrent control group, and instead the results were compared to data from the National Institute of Neurological Disorders and Stroke (NINDS) r-tPA trial. Patients with probability of AIS were also included. The null hypothesis with the preset level of significance (alpha = 0.10) for continuation in a randomized controlled study was just barely met.  Table 3 provides details about clinical trials investigating the neuroprotective drugs.

## 6. Neurogenesis

### 6.1. Granulocyte Colony-Stimulating Factor (G-CSF)

Granulocyte colony-stimulating factor (G-CSF) is a growth factor cytokine and hormone that stimulates the bone marrow to produce granulocytes and stem cells. Genetically engineered recombinant human G-CSF (such as leucostim and filgrastim) is frequently used in the treatment of neutropenia associated with chemotherapy or bone marrow transplantation. Their physicochemical characteristics and specific biological activity are equal. Preclinical results from numerous studies in stroke models statistically confirmed that recombinant G-CSF has both neuroprotective and neuroreparative effects, activating antiapoptotic, antioxidative, and anti-inflammatory signaling pathways, and stimulating angiogenesis [45]. For further translation into the clinical setting, several safety and feasibility studies were conducted using different dosages and different recombinant G-CSF analogs as additional treatment in AIS patients.

A Russian research group evaluated leucostim effects on blood cell count after AI and specifically focused on leukocytes and progenitor stem cells. In their randomized controlled study leucostim was given s.c. 10 mg/kg/day in addition to conservative treatment. They concluded treatment within 48 hours after AIS for 5 days to be safe, with no significant difference in the outcome (NIHSS, BI, Glasgow Outcome Scale) compared to the control group shown [25]. The study group was small with only 20 patients enrolled, and only six patients completed the full G-CSF treatment. The treatment was given in addition to conventional therapy, but patients with thrombolysis were excluded from this study. Also, there was no placebo given to the control group. For the safety analysis all patients were included, but for the efficacy analysis only the patients who completed the 180-day follow-up were included.

A Chinese randomized controlled trial was conducted on ten patients with middle cerebral artery infarction. Seven were treated with filgrastim 15 *μ*g/kg/d s.c. for 5 days. Outcome was assessed with the National Institute of Health Stroke Scale (NIHSS), European Stroke Scale (ESS), European Stroke Scale Motor Subscale (EMS), and Barthel Index (BI). Initial treatment within a window of 7 days demonstrated beneficial effects on outcome compared to nontreated patients, while G-CSF administration within 24 hours after stroke was superior. Furthermore, every patient underwent MRI and PET at the 12-month follow-up, showing increased metabolic activity in the peri-infarction area after G-CSF treatment [21]. There was no mention of whether thrombolysis was performed in these patients. This study did not prespecify a baseline infarction volume.

In Germany, 20 patients with AIS were enrolled in an open-label dose-escalation study and treated with three dosages of filgrastim s.c. (2.5, 5, or 10 *μ*g/kg/daily for 5 days) within 12 hours of onset [46]. Full standard care was given, including thrombolysis with i.v. r-tPA (0.9 mg/kg) within 3 hours of stroke. Although the inclusion criteria specified ischemia in the middle cerebral artery (MCA) territory, one patient with anterior cerebral infarction and two patients with vertebrobasilar territory infarction were regarded as a minor protocol violation and not excluded. Also, because the age window was broad (age over 18 years), two patients in their thirties were included. In small study groups this can have a big impact. Infarction area was evaluated with voxel-guided morphometry and neurological outcome was assessed (NIHSS, mRS, BI). Neurophysiological testing showed a time-dependent improvement, but there was no comparison to a control group. Four patients with adverse side events were suggested to be unlikely related to G-CSF.

In a Japanese prospective phase I study, filgrastim was given to 18 patients in three doses i.v. (150, 300, or 450 *μ*g/kg/day) at two time points after event (24 hours or at 7 days). The lower dosages produced no increase in leukocytes and were safe and well tolerated. Past 90 days, neurological outcomes (NIHSS, mRS, BI) were improved in those with G-CSF treatment within 24 hours compared to treatment starting 7 days later [22]. This study is limited due to the small numbers of patients (*n* = 3 for each group and time point). The aim to evaluate two treatment windows and different dosages in a single phase I study with only 18 enrolled patients may have been too optimistic. Additionally, the conventional stroke therapy regimen such as antiplatelet or anticoagulant agents in the acute phase differed from the subacute phase group.

Two German research groups conducted two multicenter trials with large cohorts. The first was the AXIS-2 trial that enrolled 44 patients in a randomized, placebo-controlled dose-escalation study analyzing four i.v. dosages of filgrastim over 3 days (30, 90, 135, and 180 *μ*g/kg cumulative doses). The treatment window was 12 hours after stroke [23]. Elevation of leukocyte counts never reached the prespecified level for termination and decreased spontaneously at the end of therapy; however, no harmful effects were observed at the 3-month follow-up. These results led to the subsequent multicenter, randomized, and placebo-controlled trial with a cohort of 324 treated patients. Filgrastim was administered within 9 hours after the stroke event in a cumulative dose of 135 *μ*g/kg i.v. over 72 hours to patients with medium and large ischemic infarctions in MCA territory. A tendency for radiologically reduced infarction volume was observed, but the study failed to prove significant beneficial effects in outcome (NIHSS, mRS, BI) after 3 months [24], despite promising preclinical and clinical data. These results demonstrate the challenges in translating findings from the animal laboratory to clinical stroke patients.

In summary, there is no clinical trial data published to date that shows significant successful effects of G-CSF treatment in a large cohort of stroke patients. Leukocyte counts were temporarily increased during treatment, yet no harmful effects were observed in any trial. Mobilized hematopoietic cells in peripheral blood were elevated after recombinant G-CSF treatment, proving effects of treatment. Early administration of G-CSF treatment of AIS within 12 to 24 hours after onset has only shown a trend towards beneficial effect on outcome.

## 7. Neuroinflammation

### 7.1. SA4503

Sigma receptors are involved in several central nervous system (CNS) disorders. More specifically, sigma-1 receptors found in the endoplasmic reticulum are binding sites which may have an effect on neurotransmitter systems via calcium signaling. Cutamesine is a ligand selective for this receptor and may oversee some neuroprotective effects in the framework of neurodegenerative diseases [47]. A growing body of evidence indicates the involvement of sigma-1 receptors in the mechanisms of various therapeutic drugs; examples include donepezil and neurosteroids [48].

Previous studies prove that IL-1*β*, TNF-*α*, and IFN-*γ* levels are elevated in the ischemic hemisphere following stroke. Sig-1R activation reduces microglia activity and release of TNF-*α*, IL-10, and nitric oxide in lipopolysaccharide activated cells. It also helps to stabilize some intracellular proteins in response to cellular stress and induces bcl-2 in reactive oxygen species dependent apoptosis [49]. Ruscher and colleagues demonstrated that treatment with SA4503 in a rat MCAO model had no effect on proinflammatory cytokines in the infarct core or peri-infarct region but resulted in a significant increase in Iba1 expression in the infarct core [50].

Urfer and colleagues conducted a multicenter, randomized, double-blind, placebo-controlled phase II study with 60 patients giving once daily low and high dose oral cutamesine treatment 48–72 hours after AIS for 28 days. It was reported that the average time from stroke onset to starting treatment was 60 hours. At the end of the 28th and 56th day there was no significant neurological improvement between low and high dose regimens compared to the placebo group. However, patients with a baseline NIHSS score of ≥7 and 9 showed a statistically significant difference between the 3 mg/d cutamesine group and placebo from baseline total NIHSS at the end of treatment [49]. The authors pointed out that patients treated with 3 mg/d cutamesine had a better 10-minute walk test compared to placebo at the 28th and 56th day but with no statistically significant difference, even though these patients received similar hours of daily rehabilitation therapy. In this clinical trial, treatment was started in the subacute phase of stroke. Consequently, the patient population included mostly neurologically stable patients compared to those in the acute phase. Treatment was applied long after thrombolytic therapy; thus patients had higher baseline neurological scores before initiation of oral cutamesine. This likely explains why oral cutamesine treatment has better outcomes in patients with higher baseline neurologic score. The results of this trial might support the use of cutamesine as a supportive drug along with physical therapy in patients who have moderate neurological status after AIS.

In the following sections, we will discuss different groups of neuroprotective agents according to their mechanism of action such as excitotoxicity and oxidative stress reduction.

## 8. Excitotoxicity

### 8.1. Caffeinol

Caffeinol includes caffeine plus ethanol and acts through central adenosine, gamma-aminobutyric acid (GABA) A, and N-methyl-D-aspartate (NMDA) receptors [51]. Belayev and colleagues applied caffeinol in ischemic rats starting at 15 minutes after reperfusion with a 2.5-hour infusion. They reported a significant decrease in cortical infarct volumes and an increase in neurological scores; however, no significant difference was found on subcortical infarct volumes and brain edema [52]. Zhao and colleagues applied caffeinol to rats up to 2-3 h after the onset of transient focal ischemia and found a dramatic decrease in cortical infarct volume [53]. This in vivo excitotoxicity model based on intracortical infusion of NMDA and a model of reversible focal ischemia demonstrated NMDA receptor inhibition as one of the possible mechanisms of caffeinol anti-ischemic activity. They also emphasized the antiexcitotoxic effect of caffeinol was not as potent as its anti-ischemic effect. Conversely, in a rabbit small clot embolic stroke model, caffeinol treatment was administered as an infusion or as multiple bolus injections but no improvement in behavioral rating scores following an embolic stroke was detected [54]. Lapchak and colleagues explained that the conflict regarding rat studies is due to the fact that ischemic lesion in this rat model not only is restricted to the cerebral cortex but also includes subcortical regions. This observation is compatible with the study by Belayev and colleagues, which found no caffeinol neuroprotective effects on subcortical infarct volumes. In the same study, Lapchak and colleagues combined caffeinol administration with low-dose t-PA. However, this combination reduced neurologic scores and raised the incidence of intracerebral hemorrhage, although not significantly.

Martin-Schild and colleagues designed a phase I, nonrandomized, single group assignment trial with 20 patients and aimed to investigate if caffeinol (caffeine 8-9 mg/kg + ethanol 0.4 g/kg IV X 2 h, started 4 hours after the onset of symptoms) and hypothermia (starting 5 hours after symptom onset, continued for 24 hours reaching a target temperature between 33 and 35°C, and followed by 12 hours of rewarming) could be administered safely together with t-PA treatment in first 3-4 h of acute ischemic stroke. Neurologic improvement in NHISS scores and adverse events related to t-PA was not higher than the expected rate. Main weaknesses of the trial include inadequate number of patients, no placebo group, and only about 1/2–2/3 of the patients being reported to have reached the targeted post-caffeinol caffeine and ethanol levels used in previous rodent studies [17].

## 9. Oxidative Stress and Cytoprotection

### 9.1. Edaravone

Edaravone (3-methyl-1-phenyl-2-pyrazolin-5-one) is a radical scavenger that inhibits nonenzymatic lipid peroxidation and lipoxygenase pathways. It has neuroprotective roles against ischemia or reperfusion-induced vascular endothelial cell injury as well as against delayed neuronal death, brain edema, and neurological deficits. Edaravone was shown to avoid extravasation of r-tPA administered in an ischemic stroke rat model thereby reducing the incidence of hemorrhagic transformation. In an observational study, edaravone treatment was found to have a low frequency of hemorrhagic transformation and mortality. In a propensity-matched analysis study, Wada and colleagues reported that combining r-tPA treatment with edaravone improved mRS scores at discharge after acute ischemic stroke. No significant effect was found on 7-day mortality, hemorrhagic transformation, or length of hospital stay [55].

An ongoing phase II clinical trial (NCT02430350) is focusing on different doses of edaravone injection for 14 days following AIS. The investigators are evaluating neurological outcomes, cognitive assessment, and safety of the drug out to 90 days. In Japan a regimen combining r-tPA + MCI-186 has been used with 30 mg i.v. MCI-186 over 30 minutes twice per a day for 14 days following AIS.

Kaste and colleagues conducted a phase II multicenter, double-blinded, placebo-controlled clinical trial in 36 ischemic stroke patients. Two different doses at 24 h after stroke were planned. Various acute adverse effects were reported in 88.9%. Hypertension was found to be the most frequent hemodynamic adverse effect in both groups. Atrial fibrillation was more frequent in high dose treatment group but there was no significant difference for other adverse effects between the 2 dose regimens. Both dosing regimens raised the plasma concentration to a plateau within 24 hours; however, this trial was lacking in detailed pharmacokinetic parameters. The authors analyzed neurological impairments in the full analysis population of which all patients had a baseline NIHSS score of 3–15. There were no significant differences in neurological improvement results between the placebo group and the 2 dosing regimens at 72 h, 120 h, 31 and 87 days [18]. The main limitation of this trial is inadequate patient enrollment compared to 252 patients that enrolled in the Japanese r-tPA + MCI-186 study. Furthermore, since Kaste and colleagues reported no significant acute neurologic improvement at the end of the treatment (72 h) after stroke, two possible perspectives can be considered. First, acute MCI-186 infusion might have better clinical effects in the chronic period (more than 3 months) after stroke and it deserves investigation. Second, researching the effects of long term MCI-186 treatment following acute ischemic stroke (14-day treatment) as done in the Japanese drug approval protocol seems warranted. Furthermore, due to high acute adverse effects, oral treatment regimens might be more feasible than i.v. infusion in clinical trials.

### 9.2. Glyburide (RP-1127)

Glyburide (RP-1127), also known as Glibenclamide (Gbc), is an antidiabetic drug in the class of sulfonylureas and functions by blocking either adenosine triphosphate- (ATP-) sensitive K^+^ channels and/or sulfonylurea receptor 1 (SUR1) in various experimental ischemic models [56]. KATP channels are rapidly activated in response to an increase of intracellular ADP/hypoxia causing K+ efflux that leads to inflammation and oxidative stress. Blockade of sulfonylurea receptors with low doses of Gbc reduced cerebral edema and infarct volume and decreased mortality by 50% in ischemic stroke rat models. Sulfonylurea receptors are associated with the astroglial NCCa-ATP channel [57] and microglial KATP channel. Abdallah and colleagues exhibited Gbc effects on diminishing neutrophil recruitment, recovering prooxidant/antioxidant balance, decreasing inflammatory mediators, increasing IL-10, and mitigating reperfusion-induced hypoglycemia in a study using Gbc pretreatment and Gbc 10 minutes before ischemia-reperfusion [58].

Sheth and colleagues conducted an open-label, single group assignment, phase II trial (GAMES-Pilot) in 10 severe anterior circulation ischemic stroke patients, 90% of whom had high NIHSS scores and received r-tPA. They aimed to investigate feasibility and tolerability of RP-1127 in this setting. The study results included reduced hemispheric volume, hemorrhagic transformation, lesion growth, and mortality, along with improved neurological outcomes. Limitations to this trial include small study size and no placebo control group [59]. The same group conducted a retrospective, case-control clinical trial using patients from the GAMES-Pilot trial, using matched control cohorts who were in two different stroke protocols at a single institute. They aimed to evaluate the effect of i.v. Gbc on vasogenic and cytotoxic brain edema with MMP-9. No difference between apparent diffusion coefficient (maps sensitive to cytotoxic edema in early stages of infarction) values at day one was found, but lower MMP-9 levels were detected in i.v. Gbc patients [59]. As the study highlighted, the main disadvantages in this trial are the insufficient number of patients with different baseline data between control groups and timing heterogeneity for MRI and blood sampling between groups. In addition, the GAMES-Pilot study included only large hemispheric infarction and it is challenging to adapt these results to ischemic stroke patients. On the other hand, it is important to emphasize that i.v. Gbc treatment was associated with lower hemorrhagic transformation and MMP-9 levels.

A recent and more promising GAMES-RP trial with the same research group was designed as a randomized, prospective, placebo-controlled, double-blind phase II trial of RP-1127 in subjects with a severe anterior circulation ischemic stroke. Important criteria for inclusion are patients with r-tPA treatment and 10-hour treatment window for i.v. Glyburide injection. The author's rationale was due to the GAMES-Pilot study data that revealed that RP-1127 had positive effects up to 10 h. The pivotal objective of GAMES-RP study is to specifically purpose patients who are prone to malignant edema after ischemic stroke.

### 9.3. 3K3A-APC

Activated protein C (APC) is derived from plasma protease zymogen, provides neuroprotection, and has antithrombotic and anti-inflammatory effects. APC stimulates multiple cytoprotective pathways via the protease activated receptor-1 (PAR-1) reducing ischemia induced injury [60]. 3K3A-APC is a recombinant variant of human APC that was designed to preserve activity at PAR-1, such as cell signaling actions, but with reduced anticoagulation. Although reduced anticoagulation may seem counterintuitive, when APC was used for the treatment of sepsis, serious bleeding was a common side effect. Thus, a modified APC (3K3A-APC) was created to address this issue. A phase I trial [26, 61] characterized pharmacokinetics and anticoagulation effects demonstrating that this protein was well tolerated at multiple doses as high as 540 g/kg 2x daily for 3 days. In addition, it also confirmed the drug had minimal coagulopathy effects. The study established tolerability and safety of the 540 mg/kg dose as only nonserious side effects were observed. 3K3A-APC shows synergistic efficacy in combination with r-tPA, with hemorrhage rates reduced after r-tPA treatment in combination [62–64]. However, when used in combination, 3K3A-APC shows no effect on r-tPA lytic effect [65].

3K3A-APC was shown to protect aging female mice as well as comorbid spontaneously hypertensive rats from ischemic stroke, as well as to extend the therapeutic window of r-tPA [61]. While all of this data is reassuring, there still remains uncertainty regarding whether the drug will work in humans, as there is no valid guide to translate effective serum concentrations in rodents to dosing in humans, as highlighted by the authors. In the past, many neuroprotectant agents have failed partly because serum concentrations were significantly lower in subjects than in the successful animal studies. It is reassuring that the maximally tolerated dose in healthy volunteers is well above the dose (200 g/kg) shown to be maximally protective in animals [66, 67].

There is currently an ongoing phase II trial, RHAPSODY, to evaluate the safety and efficacy of 3K3A-APC in multiple intravenous doses. This double-blinded, randomized study aims to measure adverse events that meet dose limiting toxicity, as well as the maximum observed plasma concentration of the target drug. Participants suffering from ischemic stroke will be treated with 3K3A-APC or a placebo, as well as either r-tPA or mechanical thrombectomy, or both treatments. The RHAPSODY trial is scheduled to be completed in March 2017 (NCT02222714).

Further clinical development should include a tolerability study in stroke subjects [26, 61]. In summary, the molecule demonstrates it was engineered for minimal coagulopathy.

Table 4 provides details about clinical trials investigating the drugs.

## 10. Conclusion

In this review, a number of clinical trials resulting in positive findings were identified. These hold the potential for future phase 3 studies in the area of AIS. The combination of eptifibatide with r-tPA has brought great hope to the future treatment of AIS. The “CLEAR” trials proved safety and tolerability up to 36 hours with dose escalation. Studies on reteplase with abciximab provided optimistic results regarding the possibility of increasing reperfusion after AIS. Furthermore, reteplase has joined the list of drugs being considered for use in the clinical setting. There is potential for synergy of drugs that inhibit platelet adhesion to the subendothelium and thrombolytic agents thereby improving clot degradation.

A very common issue is the challenge of enrolling a sufficient number of representative patients with comparable characteristics. Therefore, the results represent more a preliminary estimation than a representative evaluation. Some trials lack a control group. As in many stroke trials NIHSS, mRS, and BI evaluation does not necessarily correspond with infarction volume and overall tissue damage. Furthermore, NIHSS scores at presentation should be similar in each arm, as it is a major contributor and predictor of clinical outcomes.

When considering the results of the phase I/II clinical trials discussed in this review, many challenging unanswered questions still remain. Specific drugs offer higher potential for clinical translation, including revacept and eptifibatide. The pursuit of a better therapy and continuation of preclinical and clinical trials is imperative in order to reach a new level of treatment options for acute ischemic stroke. Future phase 3 clinical trials will be observed closely to determine which of these therapies hold the key to advances in treating AIS.

It is important to highlight that neuroprotective treatment strategies after acute ischemic stroke should focus on the progression of destructive molecular and biochemical cascades following cerebral hypoperfusion and anaerobic glycolysis. The aims of these neuroprotective approaches are to prevent ischemic brain injury from progressing into infarction. The ischemic penumbra is a conceivably recoverable region around the ischemic core where collateral cerebral blood flow supports neuronal perfusion. Thrombolytic and endovascular treatment provides reperfusion and attenuates the development of irreversible neuronal injury in penumbral region. Ischemic cascades have various spots in which its inhibition might increase the effectiveness of perfusion with thrombolytic and endovascular strategies. Time window is one of the crucial factors determining the treatment efficacy, particularly in thrombolytic and antithrombotic drugs. When eptifibatide was applied within 3 hours and reteplase + abciximab within 3 to 6 hours, neurological improvement was reported. Similarly time windows in recent endovascular clinical trials treatments are from 6 to 12 hours.

Possible pathophysiological steps that can be manipulated following anaerobic glycolysis are ionic imbalance, oxidative effects of free radicals, excitotoxicity, neuroinflammation, and apoptosis. The neuroprotective drugs are generally applied 24 to 72 hours after ischemic stroke onset and have favorable outcome in trials. This is due to the fact that their mechanism does not depend on restoring cerebral perfusion and therefore differs from antithrombotic and fibrinolytic drugs. Thus, encouraging treatment strategies should focus on combination therapies with fibrinolytic drugs in early stroke and neuroprotective drugs in 24 to 72 hours.

There is growing experimental evidence regarding the association between ischemic brain injury, neuroinflammation, and endogenous neurogenesis during the recovery period. Ischemic injury stimulates neurogenesis in the subventricular zone and subgranular layer of dentate gyrus. For that reason, the recombinant form of G-CSF, by stimulating neurogenesis, is suggested to be a future additional treatment to standard of care.

Given the plethora of information from phase I and phase II clinical trials in acute ischemic stroke, it is imperative that researchers do not dismay over negative results of these clinical trials but keep looking forward to more designs and new medications used in other areas of medicine that may benefit the brain via their mechanism of action. This will not only improve health and survival but also improve neurological outcome following AIS. In addition, future clinical trials should include a larger number of patients, different time windows, fulfillment of the planned enrollment, multicenter design, application of r-tPA, use of advanced neuroimaging techniques to target penumbra, and a clear separation of patients before treatment according to neurological scores for accurate correlation between treatment and prior neurologic severity. The STAIR and RIGOR criteria should be followed.

It is well known in the literature that drug development strategies for acute ischemic stroke have failed due to incompatible preclinical models and issues with designing of clinical trials [68]. The main difficulties of drug improvement in acute ischemic stroke trials are the restricted therapeutic window before reperfusion of salvageable brain tissue, clinical misclassification, and inadequate sample size [69]. The evolving of neuroprotective agents is generally sustained by in vivo or in vitro data. Reasons of these agents success in animal studies but failure in clinical trials are due to planning and analysis problems in experimental data as well as not effective and broad usage of new neuroimaging modalities in animal studies [70]. Thus, infarct volume declines or neurologic improvements in animal models cannot be reflected into clinical trials outcomes. In order to increase the effectiveness of experimental designs researchers need to integrate the reperfusion treatments and neuroprotective agents together in animal studies with adequate sample size and more specific tissue targeting [71]. Promising future strategies might involve focusing on neurovascular unit injury, response of pericytes, and recruitment of peripheral immune cells following ischemic stroke [72].

Before starting a clinical trial, it is imperative to know the estimate number of patients and the eligible ones at each site. Main limitations for a clinical trial are age, stroke severity, time to start treatment as well as placebo control, double blind manner, and randomization mechanisms. In addition, it is time consuming, challenging, and expensive task to design, implement, and conduct clinical trials for acute ischemic stroke. For these reasons, organization of clinical trial and safety concerns is addressed to ensure the design and performance of optimal trials to safely evaluate the drug being tested. During the process of drug development, ensure that regulatory guidelines are met and the results of these studies should be revealed to the public. Thus, following Stroke Therapy Academic Industry Roundtable (STAIR) guidelines would guarantee optimal development of new acute stroke therapies follow specific recommendations that would support the translation from bench side to clinical situations [73]. All these measures would decrease pitfalls and ensure randomization and appropriate sample size and transparent reporting [74].

Over the next five years, research in therapies for acute ischemic stroke will grow along with our understanding of the various pathways in which neural injury as well as neural protection occurs. We predict that r-tPA will not be the only treatment option available for patients presenting with acute ischemic stroke.

## Figures and Tables

**Table 1:** This table summarizes the hemostatic drugs, details of clinical trials completed in AIS, route (i.v.: intravenous), phases, number of patients enrolled, and clinical trial number.

Summary of antithrombotic drug trials						
Drug	Dose	Time window	Phase	Number of patients	Clinical trial number	Citation
Eptifibatide (CLEAR)	r-tPA (0.3 mg/kg versus 0.45 mg/kg) + eptifibatide (75 *μ*g/kg bolus followed by 0.75 *μ*g/kg/min infusion for 2 hours) or r-tPA (0.9 mg/kg)	3 hours	1/2	10	NCT00250991	[3]
Eptifibatide (CLEAR-ER)	0.6 mg/kg r-tPA + eptifibatide (135 *μ*g/kg bolus and a 2-hour infusion at 0.75 *μ*g/kg per minute) versus r-tPA (0.9 mg/kg)	3 hours	2	126	NCT00894803	[5]
Eptifibatide (CLEAR-FDR)	i.v. bolus of 135 *μ*g/kg eptifibatide followed by an i.v. infusion of 0.75 *μ*g/kg/min eptifibatide for 2 hours	3 hours	2	27	NCT01977456	[6]
Revacept	Single i.v. of 10, 20, 40, 80, or 160 mg	Laboratory, clinical exams	1	30	NCT01042964	[7]

**Table 2:** This table summarizes the thrombolytic drugs, details of clinical trials completed in AIS, route (i.v.: intravenous; i.a.: intra-arterial), phases, number of patients enrolled, and clinical trial number.

Summary of thrombolytic drug trials						
Drug	Dose	Time window	Phase	Number of patients	Clinical trial number	Citation
Reteplase + abciximab	0.25 mg/kg bolus of abciximab i.v. + 0.125 mcg/kg/min infusion for 12 hours i.a. reteplase in boluses of 0.25 units (5 minutes) proximal to the thrombus + incremental doses	3–6 hours after symptom onset	1	20	FDA Protocol Number 9180	[9]

Tenecteplase	0.25 mg/kg tenecteplase 0.1 mg/kg tenecteplase 0.9 mg/kg alteplase	6 hours after symptom onset	2b	75	New Zealand Clinical Trials Registry: ACTRN12608000466347	[10]

Tenecteplase	0.25 mg/kg tenecteplase 0.9 mg/kg alteplase	4.5 hours after symptom onset	2	104	NCT01472926	[11]

**Table 3:** This table summarizes neuroprotective drugs, details of clinical trials completed in AIS, route (i.v.: intravenous; p.o.: per-oral), phases, number of patients enrolled, and clinical trial number.

Summary of neuroprotective drug trials						
Drug	Dose	Time window	Phase	Number of patients	Clinical trial number	Citation
GSK249320	i.v. escalation cohorts 1, 5, and 15 mg/kg	24–72 hours	2	42	NCT00833989	[12]
GSK249320	i.v. escalation doses of 0.04, 0.4, 1.2, 3.5, 10, and 25 mg/kg	Healthy volunteers	1	47	NCT00622609	[13]
Lovastatin	1, 3, 6, 8, and 10 mg/kg per day for 3 days	24 hours	1	33	NCT00243880	[14]
Donepezil	5 mg/day p.o. for 30 days, increased to 10 mg/day for 60 days	≤24 hours	2a	33	NCT00805792	[15]

**Table 4:** This table summarizes the different drugs, details of clinical trials completed in AIS, route (i.v.: intravenous; s.c.: subcutaneous), phases, n/a: not available, number of patients enrolled, and clinical trial number.

Summary of trials for neuroinflammation, excitotoxicity, and oxidative stress drugs						
Drug	Dose	Time window	Phase	Number of patients	Clinical trial number	Citation
SA4503	Oral treatment of 1 mg/d and 3 mg/d for a period of 28 days	48 to 72 hours	2	60	NCT00639249	[16]
Caffeinol + hypothermia	Infusion of caffeinol (9 mg/kg caffeine + 0.4 g/kg ethanol) over 2 hours	4 hours	1/2	30	NCT00299416	[17]
Edaravone	12.5 mg/37.5 mg/62.5 mg one dose every 12 hours, for period of 14 days	24 hours	2	400	NCT01929096	[18]
RP-1127	3 mg/day i.v. 3 boluses followed by infusion for 72 hours	≤10 hours	2	10	NCT01268683	[19]
RP-1127	i.v. bolus followed by continuous infusion for 72 hours	4.5 hours	2	34	NCT01132703	[20]
Filgrastim	s.c. 15 *μ*g/kg per day for 5 days	7 days	n/a	10	n/a	[21]
Filgrastim	3 i.v. doses (150, 300, or 450 *μ*g/body/day, divided into 2 doses for 5 days)	24 hours and 7 days	1	18	n/a	[22]
AX200 (G-CSF)	4 i.v. doses, total cumulative doses of 30–180 *μ*g/kg over the course of 3 days	<12 hours	2a	44	NCT00132470	[23]
AX200 (filgrastim)	135 *µ*g/kg i.v. over 72 hours	9 hours	2	328	NCT00927836	[24]
Leucostim	10 mg/kg s.c. per day for 5 days	≤48 hours	2	20	NCT00901381	[25]
3K3A-APC	3K3A-APC at 6, 30, 90, 180, 360, 540, or 720 g/kg and 5 doses: 90, 180, 360, or 540 g/kg every 12 hours after safety of the first. g/kg every 12 hours after safety of the first	Measurements at 12 and 24 hours	1	64	NCT01660230	[26]
3K3A-APC	3K3A-APC at 120 ug/kg, 240 ug/kg, 360 ug/kg, or 540 ug/kg	Measurements at 12 hours for up to 5 doses	2	100	NCT02222714	Not yet published
